# How small changes to one eye’s retinal image can transform the perceived shape of a very familiar object

**DOI:** 10.1073/pnas.2400086121

**Published:** 2024-04-15

**Authors:** Iona R. McLean, Ian M. Erkelens, Emily A. Cooper

**Affiliations:** ^a^Herbert Wertheim School of Optometry and Vision Science, University of California, Berkeley, CA 94720; ^b^Meta Reality Labs, Redmond, WA 98052; ^c^Helen Wills Neuroscience Institute, University of California, Berkeley, CA 94720

**Keywords:** object perception, cue combination, binocular vision, visual illusion

## Abstract

Vision can provide useful cues about the geometric properties of an object, like its size, distance, pose, and shape. But how the brain merges these properties into a complete sensory representation of a three-dimensional object is poorly understood. To address this gap, we investigated a visual illusion in which humans misperceive the shape of an object due to a small change in one eye’s retinal image. We first show that this illusion affects percepts of a highly familiar object under completely natural viewing conditions. Specifically, people perceived their own rectangular mobile phone to have a trapezoidal shape. We then investigate the perceptual underpinnings of this illusion by asking people to report both the perceived shape and pose of controlled stimuli. Our results suggest that the shape illusion results from distorted cues to object pose. In addition to yielding insights into object perception, this work informs our understanding of how the brain combines information from multiple visual cues in natural settings. The shape illusion can occur when people wear everyday prescription spectacles; thus, these findings also provide insight into the cue combination challenges that some spectacle wearers experience on a regular basis.

## Significance

We describe and investigate a surprising visual illusion in which humans can misperceive the shape of a highly familiar object: their own mobile phone while they hold it in their hands. Unlike many other illusions that rely on sparse visual information, this shape illusion is robust in a fully natural environment. Our results suggest that this illusion results from a failure of the visual system to discard a single distorted visual cue. This failure informs our current understanding of sensory cue combination in natural settings and highlights the many factors that govern how we integrate information from multiple, potentially conflicting, sensory cues.

Even under the best of circumstances, vision provides ambiguous information about the geometric properties of objects. For example, a change in the retinal image of an object can be caused by a change in that object’s shape, pose, or both. As such, an important stage of visual processing is to combine information across multiple cues to determine the best guess about the geometric properties of the world. This cue combination increases the precision of sensory estimates, resolves ambiguities, and facilitates a stable neural representation of objects (1–4). As a result, in daily life, our percepts of object shape seem to be stable and relatively veridical. Here, however, we describe and investigate an illusion in which cue combination appears to distort the perceived shape of a real, familiar object. We leverage this illusion to better understand how the visual system merges multiple cues, along with past experiences, to determine the geometry of objects in the world.

The illusion we study arises when one of the eye’s images is slightly magnified. This scenario has long been a topic of perceptual investigations because it can occur when people wear prescription spectacles with unequal power between the eyes (5–14). Prescription spectacles that cause perceptual distortions and visual discomfort may lead people to eschew vision correction, so understanding this illusion is of both theoretical and practical importance. Previous research on this topic has focused on using controlled visual stimuli to investigate how interocular image size differences can distort the perceived pose of surfaces—specifically, their perceived slant. This change in perceived slant can be mathematically explained by the alterations that interocular image size differences cause to binocular disparities (i.e., the differences between the left and right eye’s retinal images) (13).

During our own research on this topic, however, we observed informally that under natural viewing conditions, many observers were unaware of any slant distortion. Instead, they were more disturbed by a salient but poorly understood illusion of object shape (7, 9–12, 14, 15). Specifically, when looking at rectangular objects, participants reported that one side appeared taller than the other. Here, we first report an investigation that aimed to capture the consistency and magnitude of this shape illusion with real objects. Our investigation further confirmed that percepts of distorted object slant were weaker and less consistent than the shape illusion. Next, we report a controlled perceptual study showing that this shape illusion is linked to distorted binocular disparity cues for object slant, even if the distorted slant does not reach awareness during natural viewing. The inability of the visual system to disregard distorted visual cues in the presence of multifaceted sensory information and prior knowledge suggests a compelling constraint on how the visual system represents the shapes of objects.

## Results

### Monocular Retinal Image Magnification Produces a Strong Shape Illusion Under Natural Viewing Conditions.

**Fig. 1.**
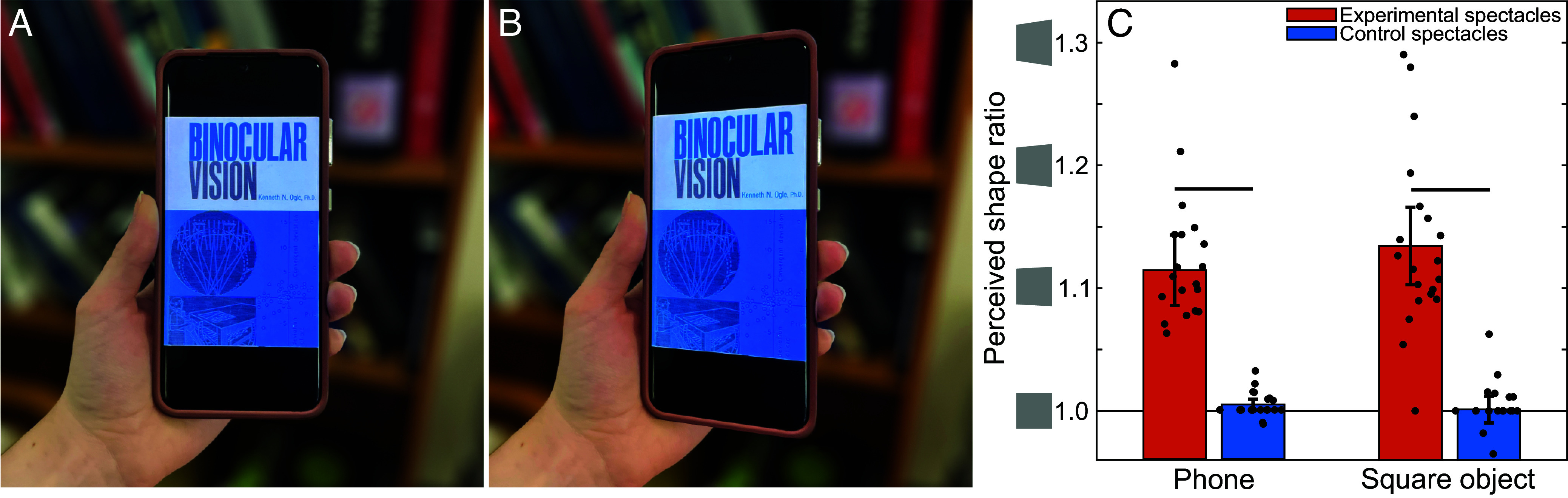
Spectacles with a monocular horizontal magnifier cause real objects to appear distorted under natural viewing conditions. (*A*) An image of a mobile phone held frontoparallel to the camera was warped to match the average shape ratio that participants drew when wearing the control spectacles (plano lenses). (*B*) The same image was warped to match the average shape ratio that participants drew when wearing the experimental spectacles with a monocular horizontal magnifier. The increase in length on the right side is equally split between the bottom and top right corners; however, participants varied in whether they saw equal or unequal stretching of top right and bottom right corners of their phones. (*C*) Bar heights indicate the average shape ratio: the ratio of the length of the right side of participants’ drawings to the left side. This includes drawings of their own phone (*Left*) and an unfamiliar square object (*Right*). The black dots in the figure represent each participant’s shape ratio. Ratios greater than 1 indicate that the right side was drawn taller than the left side, and ratios less than 1 indicate that the left side was drawn taller than the right side. Error bars represent the 95% CI and horizontal lines represent significant differences. If we assume that perceived shape is determined based on the binocular disparities created by the spectacles, and assume a typical viewing distance of 35 cm, we expect participants to see a shape ratio of 1.05 with the experimental spectacles on.

We first aimed to quantify the shape illusion under natural viewing conditions of real objects, with rich sensory cues and prior knowledge. Thus, we asked participants to hold their own mobile phone in their hand and look at it through either a pair of control spectacles (plano lenses in front of both eyes) or experimental spectacles (a plano lens over the left eye and a 3.8% horizontal magnifier over the right eye). Then, participants removed the spectacles and drew the shape that they perceived the phone to have. Illustrations of the average resulting shapes are shown in Fig. 1*A* (control spectacles) and *B* (experimental spectacles). As expected from our informal observations, the experimental spectacles elicited a strong and consistent illusion of a trapezoidal shape.

We quantified the magnitude of the illusion as a shape ratio: the ratio between the length of the right side and the left side in each drawing. When participants wore the experimental spectacles, they systematically drew the right side of their phones taller than the left side, but they did not do so for the control spectacles (Fig. 1*C*, left bars). We wondered whether this illusion might be even stronger for an unfamiliar object, so participants also performed the same task with a small, textured plastic square (that is, a flat rectangular prism with a square-shaped face). We found that the shape illusion was similar for this unfamiliar object (Fig. 1*C*, right bars).

A two-way repeated measures ANOVA revealed a significant main effect of spectacles (*F*(19) = 121.58, *P* < 0.001), but no main effect of object type (*F*(19) = 0.55, *P* = 0.466) or interaction (*F*(19) = 0.97, *P* = 0.337). The effect sizes associated with wearing the experimental spectacles were large for both the phone (*d* = 2.40) and the square object (*d* = 2.53).

### Hypothesized Explanation for the Shape Illusion.

**Fig. 2.**
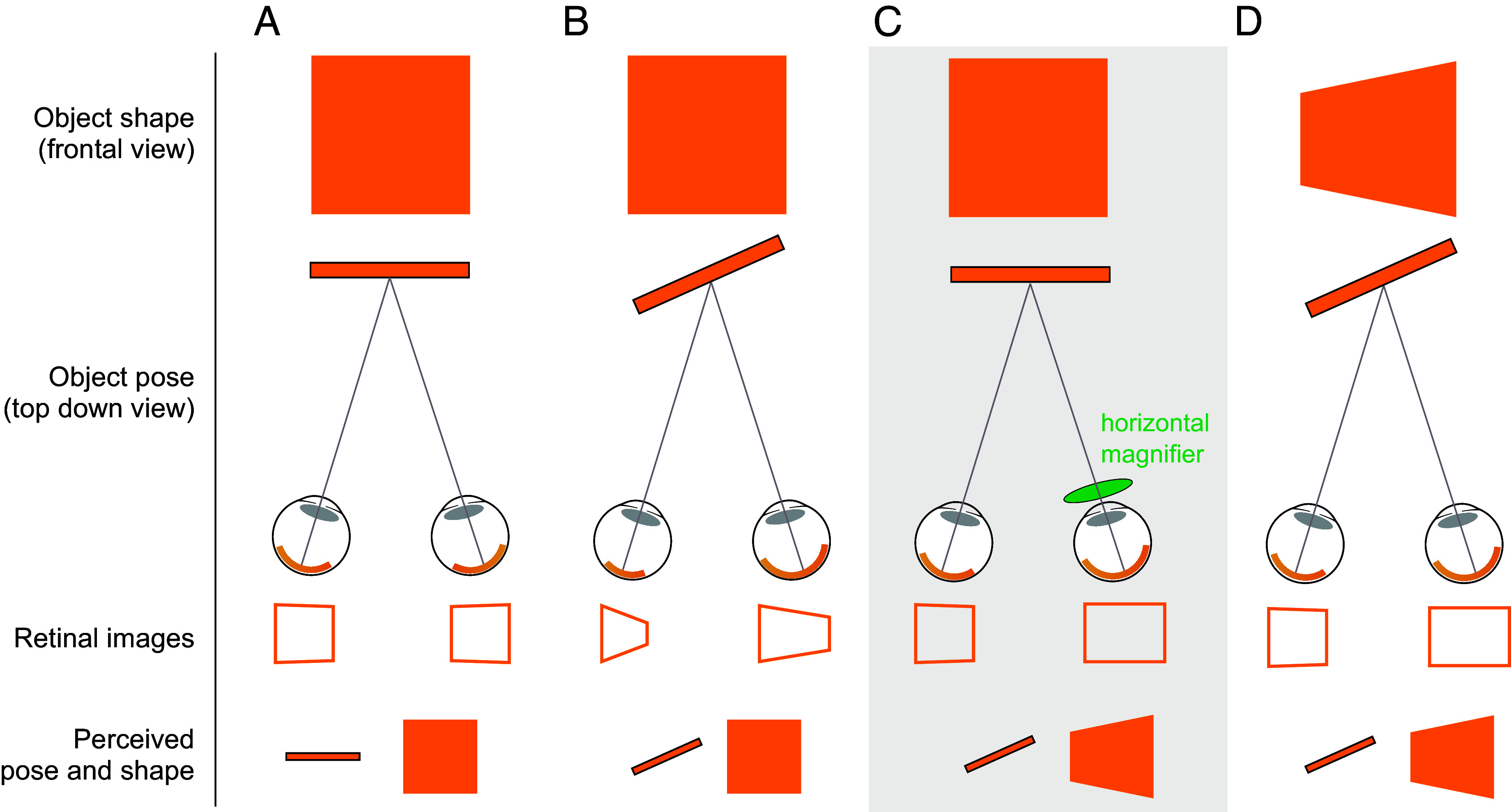
How shape and slant cues may be combined to create a perception of a three-dimensional object. (*A*) A frontoparallel square (i.e., not slanted in depth) casts roughly square-shaped retinal images, and its geometric properties (pose and shape) are likely to be perceived accurately. Small deviations from rectilinearity in the retinal images arise because each eye views the square from a slightly different angle. (*B*) A square that is slanted to face the right eye casts trapezoidal retinal images due to perspective projection. The trapezoidal retinal image in the right eye is also wider than the retinal image in the left eye, resulting in horizontal binocular retinal disparities that provide useful cues to slant angle. (*C*) If an observer views a frontoparallel square and one eye’s image is magnified slightly in the horizontal direction, this simulates the binocular disparities associated with a slanted object but does not change perspective cues. Prior researchers have shown that observers perceive the object to be slanted, but at the same time, the perceived shape becomes distorted into a trapezoid. (*D*) A trapezoid that is slanted to face the right eye can create a roughly square image on one retina and an elongated rectangular image on the other, similar to the pattern observed in (*C*).

Previous literature has posited that percepts of shape and slant are linked, which may provide an explanation for this shape illusion (10, 11, 16–19). By way of example, a square object in the world that is frontoparallel to the line of sight will project to retinal images that are roughly square-shaped, whereas a square with a pose that is slanted away from an observer will produce trapezoidal retinal images (Fig. 2 *A* and *B*). In addition, the retinal images in the two eyes are not perfectly identical, they have binocular disparities that also reflect the geometric properties of objects. If the square is slanted to face the right eye, for example, the retinal image in the right eye will be slightly wider than in the left. This creates a gradient of horizontal binocular disparities. As such, the human visual system uses information from perspective, binocular disparity, and other cues to infer the most likely three-dimensional pose and shape of an object given a pair of retinal images (20).

While the horizontal monocular image magnification produced by our experimental spectacles only slightly alters one eye’s image, this systematically changes patterns of binocular disparities. Specifically, the spectacles produce binocular disparity cues consistent with an object slanted to face the magnified eye (14), while leaving both retinal images still approximately square (one is a square, and one is a rectangle) (Fig. 2*C*). Importantly, for a flat surface to create a square retinal image when it is slanted, the object must be trapezoidal in the world. Thus, the proposed explanation for the observed shape illusion is as follows: the visual system is utilizing the altered binocular disparity cues to slant, along with the shape of the retinal images, to incorrectly infer the object’s shape (Fig. 2*D*). However, empirical studies have been inconclusive about the strength and mechanism of the link between perceived object slant and perceived object shape (16, 19, 21–24). Thus, we wondered if the participants in our study who reported the shape illusion may also be experiencing an illusory slant of the objects.

### A Slant Distortion Was also Reported, but the Effect Was Weaker and Less Consistent.

While almost all participants robustly reported a distortion in perceived shape when holding objects in their hand, pilot testing suggested that people were less confident and consistent in their experience of object slant. Thus, we created a simplified slant judgment task that enabled participants to focus on reporting just the perceived slant direction. In this task, the two objects (the mobile phone and textured square) were placed one at a time at eye height against a wall, at a similar viewing distance to the shape judgment task. Participants were first asked to report if they perceived a slant at all (that is, was the object slanted away from frontoparallel). If they said yes, we then asked them to report the slant direction (left side closer or right side closer).

**Fig. 3.**
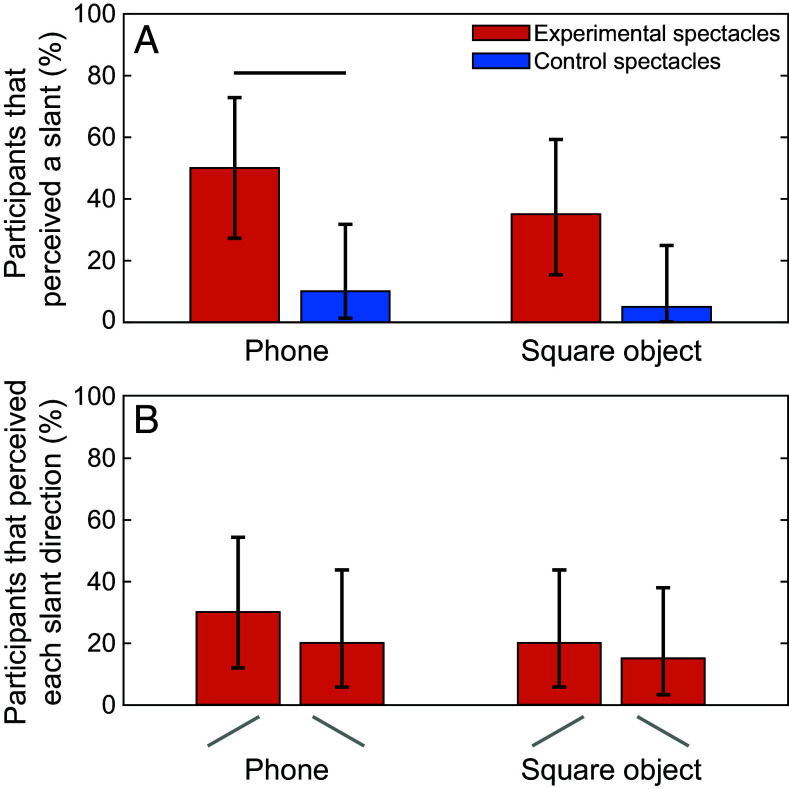
When viewing their phone and an unfamiliar square object in the experimental and control spectacles, some participants perceived a slant while others did not. (*A*) The percent of participants who perceived the objects to be slanted when placed flat against a wall and viewed through the experimental spectacles (orange) and the control spectacles (blue). Horizontal lines represent significant differences. (*B*) The percent of participants in the experimental spectacles who perceived the phone or the square to be slanted with the left side closer or right side closer. Error bars represent the binomial 95% CI.

While virtually all participants reported a shape illusion, only about half of the participants reported that the objects looked slanted through the experimental spectacles (50% for the phone and 35% for the square). This was still qualitatively more than the number who reported slant through the control lenses (10% for the phone and 5% for the square) (Fig. 3*A*). A Cochran Q test (similar to a repeated measures ANOVA, but for binary data) showed that there was a significant difference in the number of participants who perceived a slant between the conditions (*X^2^*(3) *=* 17.05, *P* < 0.001). Pairwise follow-up tests showed that a significantly greater number of participants perceived a slant when viewing their phone in the experimental versus the control spectacles (*X^2^*(1) = 6.13, *P* = 0.040, odds ratio = 0.20), but this difference was not significant when viewing the square (*X^2^*(1) = 3.13, *P* = 0.154, odds ratio = 0.14). There was also no significant difference between the two objects in the control conditions (*X^2^*(1) = 0.00, *P* = 1.000, odds ratio = 0.50) and the experimental conditions (*X^2^*(1) = 0.80, *P* = 0.445, odds ratio = 0.70).

The slant–shape relationship hypothesis predicts not just that the object should appear slanted, but that it should appear slanted in a specific direction consistent with the shape distortion (in this case, with the left side closer). Fig. 3*B* shows the breakdown of reported slant directions for the experimental spectacles. When people perceived the objects to be slanted, there was no significant difference between the direction of slant perceived (*X^2^*(3) *=* 1.46, *P* = 0.691). That is, people were similarly likely to report a slant that was consistent with the shape illusion and one that was not. This observation is in line with prior work demonstrating a tendency in some situations for people to perceived slant reversals depending on the visual cues available (10, 11).

Taken together, these findings bring into question the notion that the shape illusion observed for real objects derives from slant–shape consistency. That is, people could experience a robust distortion in perceived shape without also perceiving the slant direction that created a consistent geometric interpretation of the retinal images. However, some link between binocular disparity and shape cues still seems the most likely explanation for the shape illusion, and it has been hypothesized that slant information can influence perception outside of awareness (17, 18). Thus, we next adopted controlled stimuli to ask whether the shape illusion bears a consistent and lawful relationship to the slant specified by binocular disparities. Specifically, we leveraged the fact that horizontal and vertical monocular magnifications produce opposing slant cues in controlled settings to ask whether these manipulations also produce opposing shape illusions.

### Monocular Horizontal and Vertical Magnification Systematically Change Perceived Surface Slant in Opposing Directions.

**Fig. 4.**
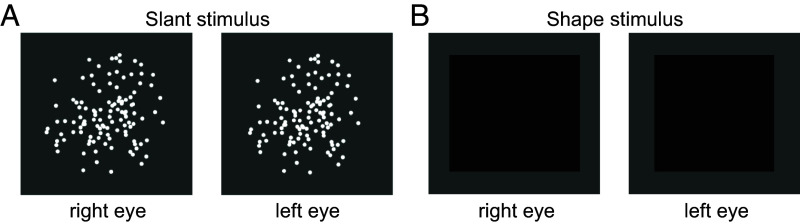
Cross-fusible stereoscopic stimuli for the slant and shape adjustment tasks. (*A*) Random dot cloud presented during the slant task. (*B*) The stimulus presented during the shape task. In each panel, the right eye’s image is horizontally magnified 6%. The dot density, dot size, and luminance in all panels have been adjusted for visual clarity. During stimulus presentation, there was a larger space (about 34 deg) between the edge of the stimulus and the edge of the screen.

In this experiment, we used controlled stimuli presented on a stereoscopic display so that we could independently change the size of each eye’s image. On a given trial, participants either adjusted the slant of a cloud of random dots until it appeared frontoparallel (i.e., not slanted, Fig. 4*A*) or they adjusted the edges of an untextured quadrilateral until it appeared square (i.e., not trapezoidal, Fig. 4*B*). The random dot cloud stimulus enabled us to measure changes in perceived slant from binocular disparity with shape cues minimized. The quadrilateral stimulus enabled us to precisely measure changes in perceived shape by focusing on the object outline. The recorded responses reflect the amount of surface slant or shape change that participants required to undo the illusion induced by monocular retinal image magnification.

**Fig. 5.**
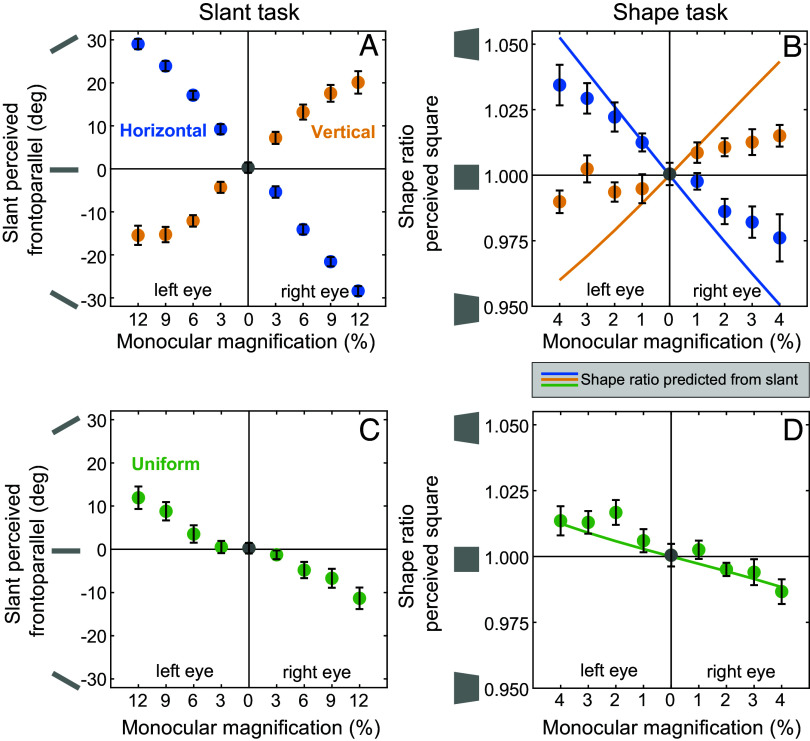
Results from the slant and shape adjustment tasks with horizontal, vertical, and uniform magnification. In each plot, circular markers indicate averages and error bars indicate the 95% CI across participants. (*A*) The slant that was perceived frontoparallel while experiencing monocular horizontal (blue) and vertical (yellow) magnification in the left eye or the right eye. The symbols beside the *y* axis indicate the direction of a positive and negative slant from a top–down view. (*B*) The shape that was perceived to be a square (specifically, the ratio between the height of the right and left side of the stimulus) while experiencing monocular horizontal (blue) and vertical (yellow) magnification simulated for the left or the right eyes. The icons next to the y axis represent shapes that would produce a shape ratio above and below 1 (the ratios are exaggerated for visibility). The lines indicate the predicted shape estimates based on the average responses from the slant judgment (fit of a third-order polynomial). (*C*) The slant percepts produced by monocular uniform magnification. (*D*) The shape percepts produced by a monocular uniform magnifier plotted in the same way as in *B*. The solid line again indicates the predicted shape estimates based on the average responses from the slant judgment.

First, we confirmed that our stimuli replicated the expected opposing slant percepts associated with vertical and horizontal monocular image magnification. As expected from previous work, we found that horizontal retinal image magnification caused participants to adjust the random dot cloud so that it was slanted to face away from the magnified eye in order to appear frontoparallel (Fig. 5*A*, blue circles). This is consistent with the notion that horizontal magnification produced a perceived slant facing toward the magnified eye. As expected, vertical magnification produced the opposite effect (Fig. 5*A*, yellow circles) (8, 14). For both manipulations, the perceived slant increased lawfully with greater magnification: across participants, we observed significant Pearson correlations between magnification and perceived slant, going in opposite directions for the horizontal and vertical manipulations (horizontal: mean *r* = −0.996 ± 0.003, *t*(19) = −750.03, *P* < 0.001, *d* = 243.34; vertical: mean *r* = 0.97 ± 0.02, *t*(19) = 120.13, *P* < 0.001, *d* = −38.97). As described by previous literature, these slant percepts can be largely explained mathematically by the change in horizontal and vertical binocular disparity cues and have been called the geometric and induced effects, respectively (8, 14). Further, when magnification was in the vertical direction, the perceived slant plateaued at higher magnitudes as compared to horizontal magnification (8).

### The Shape Illusion Correlates with the Perceived Slant Under Controlled Conditions.

If the shape illusion is driven by binocular disparity cues to slant, we expected participants to perceive a specific trapezoid shape such that the difference in height on the left and right sides would counteract the amount of perspective convergence associated with the perceived slant (Fig. 5*A*). To test this hypothesis, we first computed the expected shape distortion from the previously measured slant distortion for each participant. We expected participants’ responses to null the predicted shape distortion because the task was to adjust the stimulus until it was square. For these predictions, we again quantified the shape distortion as the ratio of the right side and left side of the trapezoid on the screen. These predictions are plotted as solid lines in Fig. 5*B*.

We then compared these predictions to the responses on the shape adjustment task. For this task, we used a smaller range of magnifications because higher levels often lead to double vision, which made the task challenging to complete. In general, we found that when the right eye experienced monocular horizontal magnification, the right side of the shape appeared taller compared to the left side, and when the left eye experienced horizontal magnification, the left side of the shape appeared taller (Fig. 5*B*, blue circles). Like the slant percepts, the perceived shape distortion reversed for vertical monocular magnification (Fig. 5*B*, yellow circles). Across participants, we again observed significant correlations between magnification and shape ratio, going in opposite directions for the two magnification types (horizontal: mean *r* = −0.93 ± 0.05, *t*(19) = −38.11, *P* < 0.001, *d* = 12.36; vertical: mean *r* = 0.66 ± 0.12, *t*(19) = 10.42, *P* < 0.001, *d* = −3.38).

To quantitatively compare the perceived shape to our geometric predictions (i.e., the predictions based on the data from the slant task), we calculated the coefficient of determination (*r^2^*) and the RMSE for each participant. The geometric predictions accounted for a significant portion of the variance in shape percepts for both the horizontal magnification (*r^2^* = 0.87 ± 0.07, *t*(19) = 22.77, *P* = < 0.001, *d* = −7.39) and vertical magnification (*r^2^*= 0.51 ± 0.14, *t*(19) = 7.40, *P* < 0.001, *d* = −2.40). Across participants, the RMSE tended to be small for both manipulations, although in both cases the RMSE was significantly different from zero (horizontal: RMSE = 0.02 ± 0.003, *t*(19) = 10.59, *P* < 0.001, *d* = −3.44; vertical: RMSE = 0.02 ± 0.002, *t*(19) = 19.90, *P* < 0.001, *d* = −6.46). Overall, the amount of shape distortion was less than predicted by the change in perceived slant, particularly for the vertical magnification. This distortion is also notably less than the shape distortion recorded for the real objects; however, the drawing task used in that experiment provides less measurement accuracy making comparisons across tasks challenging.

These results support the theory that the shape illusion is a result of inferences made about the combined object slant and shape that are consistent with both the binocular disparity and linear perspective in the retinal images. Under controlled conditions, these shape and slant percepts can be isolated and studied, but under naturalistic conditions, people seem largely unaware of the change in slant despite the salience of the shape illusion.

### Perceived Slant and Shape also Covary when Horizontal and Vertical Magnification Are Combined.

Since monocular horizontal and vertical magnification produce slant percepts in opposite directions, one could predict that their effects would cancel out if the retinal image is magnified equally in all directions. Indeed, from a prior geometric analysis, it is clear that equal amounts of horizontal and vertical magnification (uniform magnification) produce disparity cues consistent with a frontoparallel surface (25). Prior perceptual work, however, suggests that monocularly uniform magnification can still slightly distort perceived stimuli (6, 13). We thus asked whether uniform magnification would null both the slant and shape illusions in our stimuli. We found that uniform retinal image magnification in one eye still produced a systematic change in perceived slant (Fig. 5*C*). Subsequently, we can again ask whether the perceived shape agrees with the slant that results from the combined effects of the horizontal and vertical magnification. The data were consistent with this expectation (Fig. 5*D*): Geometric predictions for the shape percept derived from the slant responses explained a substantial and significant portion of the response variance and tended to have a small RMSE (*r^2^* = 0.65 ± 0.08, *t*(19) = 15.58, *P* < 0.001, *d* = −5.06; RMSE = 0.01 ± 0.003, *t*(19) = 7.67, *P* < 0.001, *d* = −2.49). Thus, under three different conditions of retinal image magnification (horizontal, vertical, and uniform), the direction and amount of shape distortion was well explained by the slant percepts produced by binocular disparity cues.

## Discussion

Visual illusions can surprise us, stoke our curiosity, and even cause us to question the reliability of our own eyes. However, these illusions are often achieved by creating controlled stimuli and situations that deprive our visual system of the full array of cues available during daily life. Here, we demonstrated that a small manipulation of one eye’s retinal image can robustly alter the perceived shape of a highly familiar object, viewed naturally and held in a person’s own hand. Through a combination of natural and controlled perceptual investigations, our studies demonstrate that this shape illusion is linked to distorted binocular disparity cues to object slant, even if the distorted slant does not reach awareness during natural viewing.

### Evidence of a Real-World Failure to Discard a Single Distorted Visual Cue.

If our interpretation is correct, then this illusion provides evidence for a failure of cue combination in the real world. In the research literature on cue combination, it has long been appreciated that ideal combination models should aim to incorporate as many cues as possible, while also being robust to corrupted or distorted cues (3, 7, 26). These models do a good job of accounting for the results of various controlled cue conflict experiments (27–29). However, our results reveal a scenario in which there appears to be an inability to discard a distorted cue (binocular disparity) even in a fully natural environment where there are many correct cues available to the viewer. When participants viewed their own mobile phone in their hand, they had several correct multimodal shape cues, such as linear perspective, texture gradients, motion parallax, and tactile information. They also had substantial prior knowledge about the true object shape derived from daily experience using the device, which should support accurate percepts (30). However, the current data suggest that to reconcile the distorted binocular disparity cues (indicating a pose that is slanted), observers inferred that the object they were holding was a trapezoid, violating the other shape cues and their prior knowledge.

Prior knowledge and expectations are thought to play a key role in shape perception because retinal images are ambiguous as to object shape (30). Therefore, the fact that the shape illusion was similar for an unfamiliar and a highly familiar object is surprising because it suggests that prior knowledge of the specific object did not have a notable influence on the percept. But our experiment potentially violated another prior assumption that could influence the strength of this visual illusion: temporal consistency. When participants put on the experimental spectacles, binocular disparity cues became distorted all at once. Under natural conditions, a distorted or corrupted sensory channel may be more likely to develop slowly over time, for example, due to gradual degradation of sensory receptors. Our sensory systems may therefore have had little need to evolve a short-term compensatory strategy for the sudden onset of a distorted sensory cue. Instead, long-term adaptation mechanisms may represent a more ecologically sensible approach to keep sensory systems well-calibrated. Indeed, prior research suggests that people can start to adapt to the shape distortion with a few hours of exposure (12). At the same time, some of the evidence in support of robust cue combination is derived from studies in which temporal consistency is clearly violated, suggesting that gradual change is not a prerequisite for robust cue combination (3, 28). Research that combines these cue combination models with models of statistical learning may be key for explaining cue combination in natural settings such as those investigated here (26, 31, 32).

### Violations of Slant–Shape Constancy.

The inconsistency that we observed between perceived shape and slant of the real objects suggests that people’s perceptual awareness of object shape can be influenced by cues that they are unaware of. This concept has previously been described as “registered slant,” whereby object slant information influences percepts even when people cannot perceive it (17, 18). In support of this concept, several previous studies have noted the tendency for monocular image magnification to create variable slant percepts that do not clearly link to people’s awareness of object shape (9, 11). While these discrepancies went away when we used controlled visual stimuli, the slant geometry still did not perfectly predict the shape illusion. Indeed, there is a long history of research examining the shape–slant invariance hypothesis, in which researchers have investigated whether observers make mutually consistent judgments of shape and slant under different viewing conditions (19, 33, 34). Importantly, while this previous work has established a lawful relationship between slant and shape percepts under a wide range of conditions, there is little evidence for perfect constancy in any specific viewing scenario (see ref. 35 for review).

Perceptual constancies seem to be imperfect, but our data support the notion that there is a strong and lawful tendency for binocular disparity cues to distort shape percepts. Importantly, the visual stimuli available can change the magnitude of the perceptual distortions that people experience when binocular disparity cues are manipulated (7, 8, 10, 14). Thus, while the differences in stimulus appearance in our study (random dots versus a solid shape) may contribute somewhat to the deviation we observed between the slant and shape percepts, some amount of deviation likely persists in many situations. Taken together, our findings challenge the notion that our perception of object properties is robust to corrupted sensory information under rich, multicue viewing conditions and encourages us to consider potential alternative explanations.

### Binocular Disparity May Play a Unique Role in Shape Perception.

Based on our results, we speculate that the visual system may uniquely prioritize interpretations of object shape that are consistent with binocular disparity cues, when those cues are present. For example, previous work has proposed a “primacy of stereopsis,” whereby information from binocular disparity is given privileged status during both cue combination and learning (26). The rationale is that once binocular disparities are successfully detected by the visual system, this cue provides a strong constraint on the possible object geometry. Cues that are derived from perspective, on the other hand, always rely on prior assumptions about shape and texture that may be violated. Related work on multisensory integration also supports the notion that more accurate sensory cues may have a special status in which they are used to calibrate the interpretation of less accurate cues that may drift over time (36). Thus, a selective and small distortion of binocular disparity may present a unique situation in which cue combination is simply not robust to perceptual distortions. Such primacy for binocular disparity would likely only be relevant for individuals with reliable and accurate stereopsis, and may not hold for the estimated ~7% of the population with stereoblindness (37). However, a recent study of slant perception suggested that people deemed stereoblind through clinical tests nonetheless made more accurate slant judgments under binocular viewing conditions, raising the interesting possibility that stereoblind individuals may be somewhat susceptible to this shape illusion as well (38).

At the same time, there is evidence against the notion that stereopsis places a strong constraint on perceived shape, even when people have normal stereopsis. Studies using pseudoscopic viewing devices, in which the images presented to the left and right eyes are switched, have shown that some people with normal stereopsis do not notice the change and that perceived shape distortions are relatively rare for those people who do notice a change in their vision (39–41). It is possible that pseudoscopic viewing, which presumably elicits a much stronger disparity cue conflict than a small monocular magnification, is more likely to drive the visual system to discard binocular disparity cues altogether. However, a large-scale study characterizing the experience of pseudoscopic vision in a natural environment suggests that most observers did notice something was distorted—but the nature of the perceived distortion was multifaceted and could incorporate depth reversals, size changes, and illusory surfaces (40). The existence of these diverse visual disruptions is consistent with the idea that distorted binocular disparities were influencing their percepts. However, the interpretation of these distortions in the context of natural scenery was hard to capture along a single perceptual dimension.

### Rectilinear Objects May Make Illusions More Salient, Even if All Objects Are Affected.

Monocular image magnification affects binocular disparity cues for everything that someone looks at, but we chose to focus our study on rectilinear objects because these shapes were anecdotally observed to produce the strongest illusion. For example, although someone’s own hand is presumably also a familiar shape, people did not seem to experience a large distortion of their hand while they were holding their phone. Why would that be the case? There is a long history of rectilinearity playing a key role in visual illusions. Famous optical illusions, such as the Ames room, leverage the fact that people infer that trapezoidal surfaces in a distorted room are actually rectilinear. Consistent with this notion, psychophysical studies suggest that, among the many assumptions people may make about object properties in order to interpret linear perspective, assumptions of rectilinearity prove particularly powerful for evoking three-dimensional percepts (15, 16, 19, 42). However, optical illusions often hinge on limiting the information available to the viewer from other cues. The illusion of an Ames room, for example, is easily broken if the observer is allowed to move around or view the room with both eyes. Thus, it is even more surprising that the shape illusion investigated here is robust to natural viewing conditions. This feature of the illusion makes it a unique candidate for investigating object perception and cue combination during natural viewing.

## Conclusion

From a practical perspective, the constraints and dynamics of cue combination are important to understand because some prescription spectacles wearers experience modified binocular disparities on a regular basis. The inability to get used to a new pair of prescription spectacles may result from the failure to adapt to perceptual distortions (43–45). Future research on adaptation and cue combination in natural settings may be able to support specific guidelines for mitigating nonadaptation, for example, with different lens designs or prescribing strategies (such as increasing a prescription slowly over time or instructing people to take their spectacles on and off regularly). Ultimately, a deeper understanding of how the visual system combines cues to infer the geometry of the world can support both a basic understanding of sensory processing and translational insights for people who experience alterations to their vision.

## Methods

### Participants.

Twenty adults participated in each experiment (experiment using real-world objects: 3 male, 17 female, mean age = 25.3 ± 3.7 y, experiment using simulated objects: 7 male, 13 female, mean age = 26.5 ± 4.0 y). In both experiments, inclusion criteria included normal visual acuity measured at 10 feet (20/20 binocular and 20/30 monocular equivalent or better), and normal stereoacuity (50 arcsec or better on a Randot test). The experiments were approved by the University of California, Berkeley, Institutional Review Board, and all participants provided informed consent and were compensated for their time.

### Experiment Using Real-World Objects.

#### *Tasks*.

Participants performed the same set of tasks while wearing control spectacles (a pair of plano lenses) and experimental spectacles (a plano lens in front of the left eye and a 3.8% horizontal magnifier over the right eye) (5, 12). The order in which the spectacles were worn was counterbalanced. Participants began by wearing each pair of spectacles for several minutes while exploring an indoor lab environment and looking at objects of various sizes. Then, participants performed structured observation of objects. Two objects were used: the participant’s mobile phone with text on the screen and a black 3D printed rectangular prism (H = 7.5 cm, W = 7.5 cm, and L = 1 cm) with eight randomly placed yellow dots to provide some texture. The mobile phones of all participants were rectangular, with varying aspect ratios.

The structured observation period began with the shape observation, in which participants wore each pair of spectacles while holding the objects at a comfortable viewing distance in their hand. Then, participants removed the spectacles and drew the outline of each object using a ruler. For the slant observation, participants judged the slant of each object while it was at eye height against a featureless white wall to ensure that the object was frontoparallel to the observer. Once placed against the wall, participants reported whether the object appeared slanted, and if so, in which direction. The slant and shape observations were both performed in near peripersonal space, but the distances were not precisely controlled.

#### *Analysis*.

The left and right side of each drawing was measured, and the shape ratio was calculated by taking the length of the right side and dividing it by the length of the left side. To evaluate the effect of the lenses and the objects, a 2 × 2 ANOVA was run. The ANOVA for the shape ratio did not pass assumptions for homogeneity, so we ran a permutation-based ANOVA (aovp function in the lmPerm package from R). There was no change in results, so we report the results from the original ANOVA in this paper. Cohen’s *d* was used to determine the magnitude of the effect size of spectacle type for the two different objects. For the slant data, we used a Cochran Q for the omnibus test with McNemar follow-up pairwise comparisons and an odds ratio to determine the magnitude of the effect. The Cochran Q and the McNemar tests are analogous to an ANOVA and the associated follow-up tests for binary data. The odds ratio denotes the effect size with the control group divided by the experimental group. However, when the experimental and control groups are directly compared, then the square condition is divided by the phone condition. For pairwise comparisons, *P* values were corrected using a false discovery rate of 5%.

### Experiment Using Simulated Objects.

#### *Stimuli*.

Stimuli were presented on a VIEWPixx 3D display (LCD panel with LED backlight) with a resolution of 1920 × 1080 pixels, a pixel pitch of 0.27 mm (subtending ~0.05°), and a global refresh rate of 120 Hz (VPixx Technologies, Montreal, QC, Canada). Participants wore a 3DPixx shutter glass system to view the stimuli, allowing us to independently manipulate the images shown to the left and right eyes through temporal interlacing (Nvidia, Santa Clara, CA). The maximum luminance through the right and left lenses of the shutter glasses was approximately 27 cd/m^2^. All stimuli were presented with Psychtoolbox version 3.0.18 in MATLAB (MATLAB R2022a; The MathWorks, Natick, MA). Participants sat 29.3 cm from the screen with their head on a chin rest and eyes aligned to the center of the screen. To prevent the straight edges lining the screen from being used as a reference, irregularly shaped paper was attached to the edge of the monitor.

Participants performed two interleaved tasks that measured the magnitude of the shape and slant distortions associated with monocular magnification. The stimuli were manipulated to create horizontal, vertical, or uniform magnification in just the right eye or just the left eye. Magnification was applied to one eye by changing the distance between points on the screen in one eye but not the other. There were different levels of monocular magnification in the slant task (0, 3, 6, 9, and 12%) and the shape task (0, 1, 2, 3, and 4%). These magnitudes of magnification were chosen to minimize the likelihood of diplopia (double vision) in the shape task and to be similar to the ranges in previous literature. Each condition (magnification level and eye) was repeated four times, and participants freely viewed the stimuli.

#### *Slant task*.

This task aimed to quantify the perceived slant produced by changes to horizontal or vertical binocular disparity. To measure the full magnitude of this effect without other depth cues dampening the illusion, we created a stimulus that aimed to isolate cues from binocular disparity (Fig. 4*A*). We presented a random dot stereoscopic stimulus whose slant could be adjusted without a change in shape or dot density. This way, participants could only use binocular disparity, but not shape or dot density, to make their slant judgments. This method of binocular disparity isolation is described in previous literature (46). The stimulus was a roughly 16° diameter region composed of white dots (0.05° in diameter and 100% maximum luminance) over a gray background (2% luminance). Dot density on the screen decreased from the center outward with a central 8° region having a dot density of 0.31 dots/deg^2^ and the outer area having a density of 0.062 dots/deg^2^. This tapering of density allowed us to improve cues from binocular disparity without adding strong information about the size and shape which could have been used by participants to judge slant. When magnification was applied to one of the eye’s images, this changed the distance between the dots on the screen in one of the eyes, but it did not change the dot size or shape. On each trial, participants used left and right arrow keys to adjust the slant of the circular dot cloud around a vertical axis until it appeared frontoparallel. The initial slant was randomized, and the maximum and minimum slants were also jittered.

#### *Shape task*.

The aim of this task was to quantify the magnitude of the shape distortion while encouraging participants to focus on the overall object shape. Participants used the arrow keys to adjust the y positions of the top right and bottom corners of a black quadrilateral (0.9% luminance) on a gray background (2% maximum luminance) until it appeared square (Fig. 3*B*). When the shape was a perfect square, it subtended 16° by 16°. On each trial, a random initial position and maximum and minimum adjustment was generated for the top right and bottom right corners. The shape distortion was quantified as the ratio of the height of the right side to the left side.

### *Analysis*.

We expected that the perceived shape distortion could be predicted from the magnitude of the perceived slant. Specifically, we expected that the magnitude of the shape distortion for each participant would be equal but opposite to the perspective convergence of a 16° by 16° square viewed at 29.3 cm and slanted at the magnitude of the reported slant in the slant task. We ran an analysis to compare the actual shape responses to our predictions for each participant for uniform, horizontal, and vertical magnification. We calculated a prediction for each slant data point and then fit a third-order polynomial for uniform, horizontal, and vertical magnification for each participant. Then, we calculated the *r^2^* values and RMSE for each subject. In addition, *t* tests and Cohen’s *d* were calculated to compare the *r^2^* and RMSE values for all participants against a null hypothesis of no relationship (*r^2^*= 0).

## Data Availability

Data and analysis code for both experiments and anonymized (Matlab files, Matlab code, CSV files, and R code) data are publicly available at https://dx.doi.org/10.5281/zenodo.10834940 (47).
